# Effect of roxadustat on iron metabolism in patients with peritoneal dialysis: a real-world 24-week study

**DOI:** 10.1186/s40001-023-01465-0

**Published:** 2023-11-07

**Authors:** Xuejie Zhang, Ruoyu Jia, Zhifang Zheng, Luhua Jiang, Yizhou Xu, Ashok Raj, Dong Sun

**Affiliations:** 1 Department of Nephrology, Affiliated Hospital of Xuzhou Medical University, 99 West Huai-Hai Road, Xuzhou, 221002 Jiangsu China; 2 Department of Nephrology, Jintan Hospital，Affiliated Hospital of Jiangsu University, Changzhou, China; 3 National Clinical Research Center of Kidney Diseases, Jinling Hospital, Affiliated Hospital of Medical School, Nanjing University, Nanjing, China; 4 Department of Urology, Affiliated Hospital of Xuzhou Medical University, Xuzhou, China; 5 Department of Internal Medicine and Diagnostics, Xuzhou Medical University, Xuzhou, China

**Keywords:** Peritoneal dialysis (PD), Anemia, Hypoxia inducible factor-prolyl hydroxylase inhibitors (HIF-PHI), Iron metabolism

## Abstract

**Background:**

Roxadustat is an oral hypoxia inducing factor-prolyl hydroxylase inhibitor (HIF-PHI) that regulates iron metabolism in patients with chronic kidney disease (CKD) primarily by reducing hepcidin levels and mobilizing internal iron stores. More data are needed to demonstrate the efficacy of roxadustat in regulating iron metabolism in patients with peritoneal dialysis (PD) compared with erythropoiesis stimulating agents (ESAs).

**Methods:**

This prospective cohort study enrolled PD patients with a mean hemoglobin level of 60–100 g/L. All subjects were randomized into two groups at a ratio of 2:1 the roxadustat group (106 cases), and the ESA group (53 cases). The primary endpoint was the change in the iron biomarker levels and the proportion of patients with absolute iron deficiency and functional iron deficiency.

**Results:**

Compared with ESAs, roxadustat significantly decreased hepcidin level (difference, − 20.09 ng/mL; 95% CI, − 30.26 to − 9.92), attenuated the increase in serum soluble transferrin receptor (sTFR) level (difference, − 7.87 nmol/L; 95% CI, − 12.11 to − 3.64), and reduced the proportion of patients with functional iron deficiency (roxadustat, 11.43%; ESA, 33.33%). There was no significant difference in safety of the two groups over the duration of the study.

**Conclusions:**

Compared with ESA group, roxadustat group showed significant differences in all iron biomarker levels except serum ferritin (sFt) and transferrin saturation (TSAT). These results suggest that roxadustat was superior to ESAs as a therapy for iron metabolism in PD patients.

*Trial registration*: This study completed Chinese Clinical Trial Registration on March 4, 2022 (registration number: ChiCTR2200057231).

**Graphical abstract:**

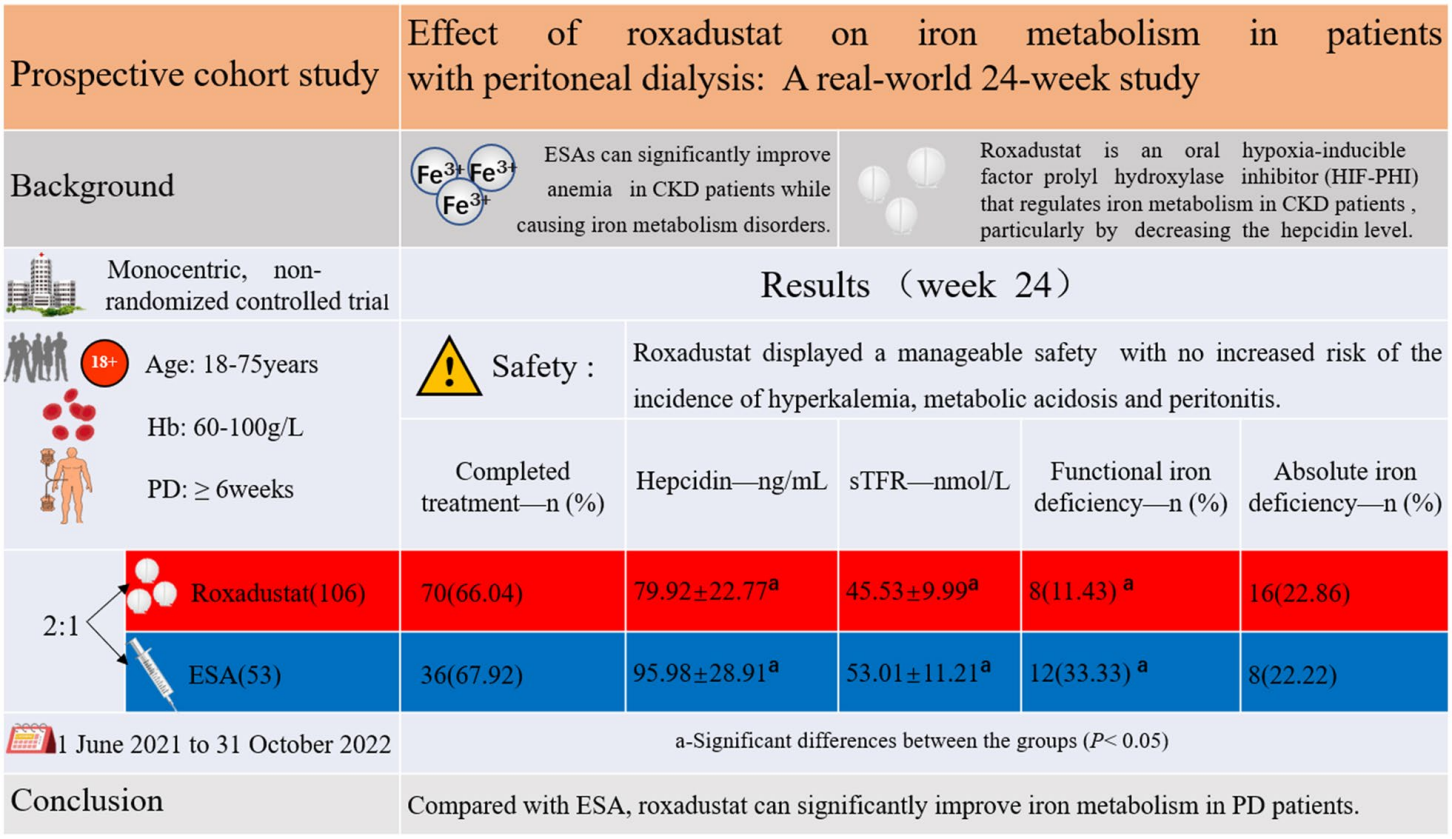

## Background

The prevalence of anemia increases with the progression of CKD [1]. Renal anemia is associated with dysregulation of oxygen sensing by the malfunctioning kidneys, leading to inadequate synthesis of erythropoietin (EPO), iron deficiency, and inflammation [2]. Iron deficiency especially functional iron deficiency due to chronic inflammation is one of the major causes of anemia in CKD patients [3]. Abnormal iron metabolism in CKD patients is mediated by hepcidin, a key regulator of iron uptake and mobilization that is downregulated in hypoxia [4, 5]. Because PD patients do not have venous blood loss problems, less clinical focus is given to anemia than hemodialysis patients, but the data suggest that among PD patients, the prevalence of iron deficiency anemia (IDA) was reported to be between 16 and 23% [6]. The inflammatory status of CKD patients changes the actual levels of these parameters by affecting transferrin and hepcidin, reducing TSAT and increasing sFt levels [7]. Therefore, sTFR, which is not affected by inflammation, may provide more information than TSAT and sFt to reflect iron metabolism in CKD patients [8, 9].

The clinical application of ESAs can increase hemoglobin concentration without the risk of transfusion-related iron overload, substantially improving patients' quality of life [10]. However, ESAs further deplete circulating iron, leading to the need for additional iron supplementation as the primary treatment for CKD anemia [11]. Meanwhile, several clinical problems have been reported, including ESAs hyporesponsiveness [12] and increased risk of cardiovascular events and death [13, 14].

By simulating hypoxic environment, roxadustat effectively inhibits HIF-PHD activity and increases the accumulation of HIF [15], thereby inducing the expression of EPO, EPO receptors, and proteins required for iron metabolism [16, 17]. The identification of HIF-PHI provides a new therapeutic means for the treatment of renal anemia [18, 19]. We now report the results of a 24-week, cohort study involving PD patients to analyze the effect of roxadustat on iron metabolism in PD patients by comparing changes in iron biomarker levels including sTFR.

## Methods

### Study design and population

This trial evaluated the efficacy and safety of roxadustat in regulating iron metabolism in PD patients through 24 weeks of observation. The eligible patients who agreed to participate in the study were numbered according to the time of visit to the hospital, and were divided into roxadustat group and ESA group with a ratio of 2:1 by random number table. This study was approved by the Ethics Committee of the Affiliated Hospital of Xuzhou Medical University (XYFY-KL39-01) and completed Chinese Clinical Trial Registration (registration number: ChiCTR2200057231), and all the enrolled patients signed an informed consent form. Eligible patients ranged in age from 18 to 75, had received stable PD for > 6 weeks, did not receive ESAs, roxadustat, and iron supplementation within 6 weeks before enrollment, and met the clinical diagnosis criteria of renal anemia as stipulated in the Chinese Expert Consensus on the Diagnosis and Treatment of Kidney Anemia (2018 Revision). Exclusion criteria included severe organic dysfunction of the heart, liver, lung, and brain, a change in the dialysis method, transfusion, and intravenous iron.

### Calculation sample size

Sample sizes (*N*1 = 34; *N*2 = 68) for the roxadustat and ESA groups were calculated using PASS 15.0 software based on a multicenter randomized controlled clinical trial conducted in China with α of 0.05 and efficacy of 1 − β of 90% [20]. A total of 159 patients were included considering an estimated drop-out rate of 30%.

### Study drug administration

Patients in the roxadustat group were treated with roxadustat capsules [Enambojin (China) Pharmaceutical Technology Development Co., LTD., Sinopharm H20180024 (50 mg), H20180023 (20 mg)], administered three times a week at 70 mg (< 45 kg), 100 mg (45 to < 60 kg) or 120 mg (≥ 60 kg). The dose was adjusted according to a preset dosing ladder as follows: 20, 40, 50, 70, 100, 120, 150, and 200 mg. The maximum dose was 2.5 mg/kg. If the patient's hemoglobin increase was greater than 20 g/L within 2 weeks and the hemoglobin value was greater than 90 g/L, the dose was lowered by one step. Only one dose reduction over 4 weeks was recommended when hemoglobin rose too rapidly. Patients in the ESA group were treated with generic epoetin alpha [Sansheng Pharmaceutical Co., LTD., S19980073 (2000 IU), S19980074 (3000 IU), S19980072 (4000 IU), S20010001 (10000 IU)] at 75–100 IU/kg/week. The maximum dose of each adjustment was 30 IU/kg/week. The initial treatment target was an increased hemoglobin level of 10–20 g/L per month, and subsequent adjustments would be made according to the patient's hemoglobin level, speed of hemoglobin change, and treatment response. Oral iron can be accepted when STAT ≤ 20% or sFt ≤ 100 µg/L while intravenous iron is not permitted during the study. The use of statins and phosphate binders was allowed.

### End points

The primary efficacy endpoints were the changes of iron biomarker levels from baseline to week 24 including hepcidin, sTFR, serum iron (SI), sFt, TSAT, total iron binding capacity (TIBC), and the proportion of patients with absolute iron deficiency (defined as TSAT < 20% and sFt < 100 ng/mL) and functional iron deficiency (defined as TSAT < 20% and sFt ≥ 100 ng/mL). Secondary efficacy endpoints were as follows: the hemoglobin levels at weeks 8 and 24, the proportion of patients who achieved the treatment target (defined as a mean hemoglobin level greater than or equal to 100 g/L but less than 120 g/L), the mean changes from baseline in lipid metabolism levels, and the incidence of hyperkalemia, metabolic acidosis and peritonitis.

### Statistical analysis

All the data were processed with SPSS 25.0. Measurement data with a normal distribution were expressed as means ± standard deviation, and comparisons between groups were analyzed using independent sample t-tests. Nonnormally distributed data were expressed as medians and interquartile ranges. The enumeration data were expressed as percentages, and the Chi-squared test was used to compare groups. We used the mixed-effects repeated-measures model to analyze the mean changes from baseline in iron biomarker levels, hemoglobin levels and lipid metabolism levels over week 24. Before analyzing the above parameters, logarithmic transformation of the values of sFt and EPO were carried out to make them meet the normal distribution. Safety was monitored by assessment of changes in blood pressure and the incidence of hyperkalemia, metabolic acidosis, and peritonitis during treatment. Bitailed *P* < 0.05 was considered statistically significant.

## Results

### Baseline characteristics of the patients

**Fig. 1:**
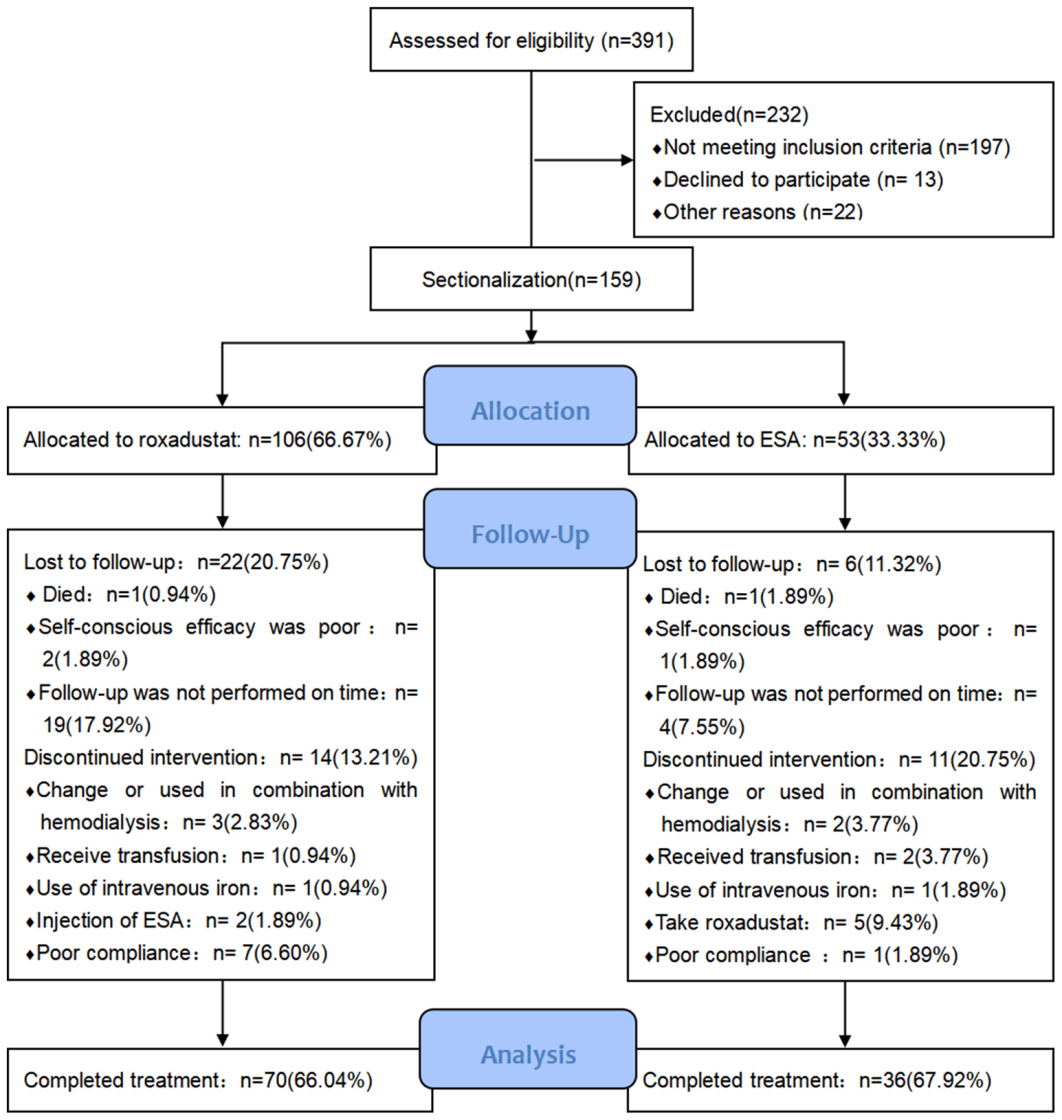
Patient disposition. Among 391 PD patients regularly followed up, 159 eligible patients were grouped [roxadustat group, *n* = 106 (66.67%); ESA group, *n* = 53 (33.33%)]. A total of 106 patients completed 24-week treatment [roxadustat group, *n* = 106 (66.67%); ESA group, *n* = 53 (33.33%)], whereas 20.75 and 11.32% of patients were lost to follow-up and 13.21%, and 20.75% discontinued intervention in the roxadustat and ESA groups, respectively

From June 2021 through April 2022, 159 patients underwent randomization (roxadustat, 106; ESA, 53). Of these patients, 106 completed the 24-week study (roxadustat, 70; ESA, 36) (Fig. 1). Median duration of PD therapy was 83.50 (40.00,120.75) and 90.00 (36.50, 209.50) weeks, respectively. The most common type of peritoneal transport was low mean transport (roxadustat, 41; ESA, 22), followed by high mean transport (roxadustat, 25; ESA, 11). The most common primary diagnoses were chronic glomerulonephritis (roxadustat, 39; ESA, 21), hypertension (roxadustat, 30; ESA, 14), and diabetes (roxadustat, 18; ESA, 9). All the patients had hemoglobin levels greater than 60 g/L and less than 100 g/L. The baseline characteristics of the patients were similar in the two groups (Table 1).

**Table 1:** Characteristics of the patients at baseline

Characteristics	Roxadustat (*n* = 106)	ESA (*n* = 53)	*P* value
Male sex, *n* (%)	59 (55.66)	27 (50.94)	0.574
Age, years	47.59 ± 13.63	50.15 ± 12.02	0.248
BMI, kg/cm^2^	23.58 ± 3.66	24.80 ± 4.01	0.056
Weight, kg	65.20 ± 12.59	66.91 ± 12.85	0.399
Duration of PD, weeks	83.50 (40.00,120.75)	90.00 (36.50,209.50)	0.207
Mean arterial pressure, mmHg	108.79 ± 17.61	105.86 ± 17.36	0.323
History of hypertension, *n* (%)	94(88.68)	45(84.91)	0.499
History of diabetes mellitus, *n* (%)	20(18.87)	10(18.87)	1.000
Peritoneal transport type:			
High transport, *n* (%)	8 (7.55)	3 (5.66)	0.970
High average transport, *n* (%)	25 (23.58)	11 (20.75)	
Low transport, *n* (%)	8 (7.55)	5 (9.43)	
Low average transport, *n* (%)	41 (38.68)	22 (41.51)	
Unknown, *n* (%)	24 (22.64)	12 (22.64)	
Protopathy:			
Chronic glomerulonephritis, *n* (%)	39 (36.79)	21 (39.62)	0.989
Polycystic kidney, *n* (%)	8 (7.55)	3 (5.66)	
Hypertension, *n* (%)	30 (28.30)	14 (26.42)	
Diabetes, *n* (%)	18 (16.98)	9 (16.98)	
Nephrotic syndrome, *n* (%)	6 (5.66)	4 (7.55)	
Others, *n* (%)	5 (4.72)	2 (3.77)	
sTFR, nmol/L	36.89 ± 10.06	36.50 ± 10.78	0.824
Hepcidin, ng/mL	104.69 ± 27.41	100.66 ± 27.68	0.385
SI, µmol/L	13.54 ± 6.46	12.80 ± 5.39	0.474
sFt, ng/mL	215.80(71.96,354.95)	195.90(83.93,354.60)	0.801
TSAT, (%)	32.13 ± 15.23	31.98 ± 14.85	0.952
TIBC, µmol/L	43.33 ± 11.44	42.44 ± 12.03	0.653
RBC, 10^12^/L	2.95 ± 0.38	2.89 ± 0.40	0.421
Hemoglobin, g/L	87.84 ± 9.42	86.66 ± 10.72	0.479
EPO, mIU/mL	6.40 (3.77, 9.05)	5.64 (3.79, 9.36)	0.949
K^+^, mmol/L	4.27 ± 0.75	4.34 ± 0.80	0.595
> 5.5 mmol/L, *n* (%)	4 (3.77)	5 (9.43)	0.145
HCO^3−^— mmol/L	21.67 ± 3.50	21.85 ± 3.59	0.774
< 22.0 mmol/L, *n* (%)	51 (48.11)	24 (45.28)	0.736
Total cholesterol, mmol/L	4.01 ± 1.20	4.23 ± 1.14	0.277
Triglyceride, mmol/L	1.54 ± 0.85	1.78 ± 1.33	0.156
HDL-C, mmol/L	0.91 ± 0.32	0.93 ± 0.30	0.686
LDL-C, mmol/L	2.43 ± 1.00	2.53 ± 0.97	0.549
C-reactive protein, mg/L	2.00 (0.50, 6.32)	2.41 (0.60, 5.80)	0.703
> 4.9 mg/L, *n* (%)	30 (28.30)	16 (30.19)	0.805
eGFR, mL/min	6.33 (4.82, 8.09)	5.48 (4.04, 7.00)	0.069
Creatinine, µmol/L	820.92 ± 216.48	883.87 ± 302.90	0.134

Measurement data with a normal distribution are expressed as means ± standard deviation, and nonnormally distributed data are expressed as medians and interquartile range. No significant differences between group were found in the baseline characteristics. BMI denotes body mass index, sTFR denotes serum soluble transferrin receptor, SI denotes serum iron, sFt denotes serum ferritin, TSAT denotes transferrin saturation, TIBC denotes total iron-binding capacity, RBC denotes red blood count, EPO denotes erythropoietin, K^+^ denotes potassium, HCO^3−^ denotes bicarbonate, HDL-C denotes high-density lipoprotein, LDL-C denotes low-density lipoprotein, and eGFR denotes estimated glomerular filtration rate

### Iron biomarker levels

During the 24-week study, 2 patients (roxadustat, 1; ESA, 1) stopped intervention because of the intravenous iron. 48 patients (45.28%) in the roxadustat group received oral iron therapy compared with 38 (71.70%) in the ESA group.

**Fig. 2:**
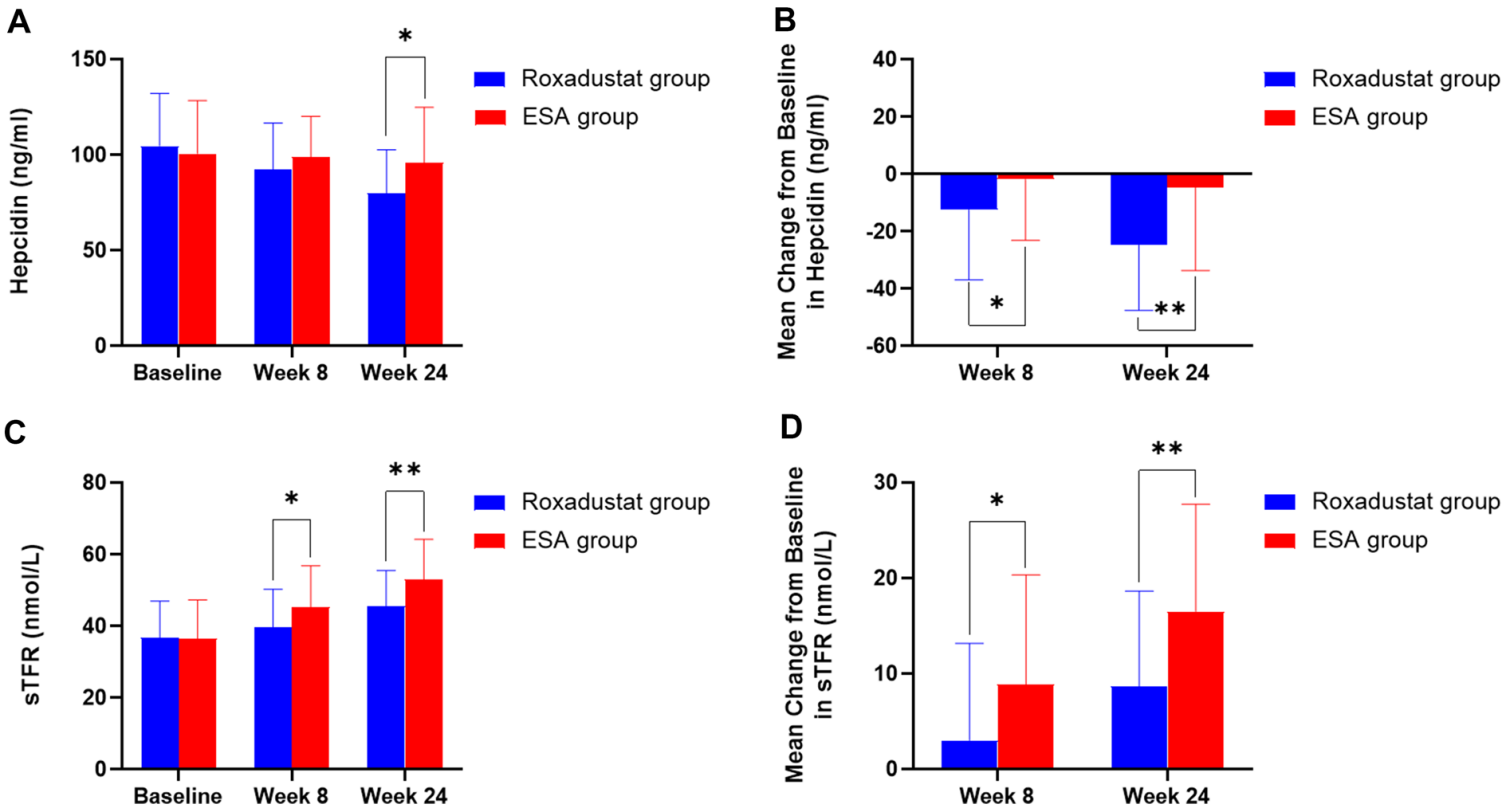
Hepcidin levels (**A**) and mean change from baseline by treatment (roxadustat versus ESAs) at week 8 and week 24 (**B**). sTFR levels (**C**) and mean change from baseline by treatment (roxadustat versus ESAs) at week 8 and week 24 (**D**). sTFR denotes serum soluble transferrin receptor. *Significant differences between groups (*P* < 0.05). **Significant differences between groups (*P* < 0.01)

At week 8, the hepcidin levels of roxadustat and ESA groups decreased from baseline by 12.46 ± 24.48 ng/mL and 1.78 ± 21.31 ng/mL, respectively (Fig. 2A). The difference between groups was − 10.68 ng/mL (95% CI, − 19.26 to − 2.11). At week 24, the change from baseline was − 24.77 ± 22.77 ng/mL in the roxadustat group and − 4.68 ± 28.91 ng/mL in the ESA group. The difference between groups was − 20.09 ng/mL (95% CI, − 30.26 to − 9.92) (Fig. 2B). There were no remarkable changes in the hepcidin levels were observed in the ESA group; in the roxadustat group, the baseline was significantly higher than week 8 (*P* < 0.001) and week 24 (*P* < 0.001). At week 8, the difference of sTFR levels between groups was − 5.86 nmol/L (95% CI, − 9.73 to − 1.99), and the additions from baseline were 3.02 ± 10.14 nmol/L and 8.87 ± 11.44 nmol/L, respectively (Fig. 2C). At week 24, the additions were 8.64 ± 9.99 nmol/L in the roxadustat group and 16.51 ± 11.21 nmol/L in the ESA group (Fig. 2D).

**Fig. 3:**
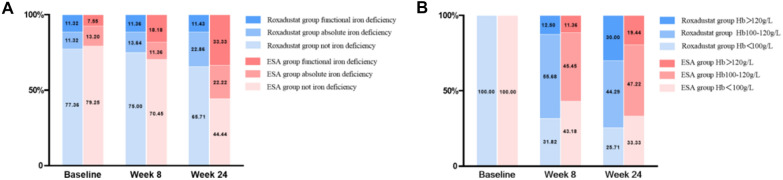
Proportion of patients with absolute iron deficiency and functional iron deficiency (**A**) and Hb < 100 g/L, Hb ≥ 100 g/L but < 120 g/L and Hb ≥ 120 g/L (**B**) by treatment (roxadustat versus ESAs) at baseline, week 8 and week 24. Hb denotes hemoglobin

In the roxadustat group, the SI and TSAT levels were clinically stable, and the changes from baseline were 0.47 ± 7.12 µmol/L (95% CI, − 1.22 to 2.17) and − 3.26 ± 15.88% (95% CI, − 7.04 to 0.53), respectively, with an increase in the TIBC, the change from baseline was 7.13 ± 9.63 µmol/L (95% CI, 4.83 to 9.42) and a decrease in the logsFt, the change from baseline was − 0.11 ± 0.40 µmol/L (95% CI, − 0.01to − 0.20). In the ESA group, the SI and TIBC were clinically stable, and the changes from baseline were − 2.11 ± 7.75 µmol/L (95% CI, − 4.73 to 0.51), and 2.81 ± 9.93 µmol/L (95% CI, − 0.55 to 6.17), with a decrease in TSAT and logsFt, the changes from baseline were − 7.85 ± 16.72% (95% CI, − 13.50 to − 2.19), and − 0.19 ± 0.43 ng/mL (95% CI, − 0.33 to − 0.04) (Table 2). At week 24, 22.86% of the roxadustat group and 22.22% of the ESA group had absolute iron deficiency, with no significant differences between groups (*P* = 0.956). At the same time, we observed that while the percentage of patients with functional iron deficiency remained stable in the roxadustat group (*P* = 0.982), ESA group increased significantly (*P* = 0.002) (Fig. 3A).

**Table 2:** Evolution of anemia and iron biomarker levels by treatment

	Roxadustat(n = 70)			ESA(n = 36)		
	Baseline (n = 106)	Week 8 (n = 88)	Week 24 (n = 70)	Baseline (n = 53)	Week 8 (n = 44)	Week 24 (n = 36)
SI, µmol/L	13.54 ± 6.46	14.54 ± 7.41^b^	14.01 ± 7.12^b^	12.80 ± 5.39	11.73 ± 5.68^b^	10.69 ± 7.75^b^
LogsFt, μg/mL	2.21 ± 0.45	2.03 ± 0.41^a^	2.10 ± 0.40^a^	2.24 ± 0.46	2.18 ± 0.38	2.05 ± 0.43^a^
sFt < 100 ng/mL, *n* (%)	32 (30.19)	40 (45.45)^a^	30 (42.86)	16 (30.19)	14 (31.81)	15 (41.67)
TSAT, (%)	32.13 ± 15.23	30.42 ± 14.17	28.87 ± 15.88	31.98 ± 14.85	27.89 ± 13.50	24.13 ± 16.72^a^
< 20%, *n* (%)	24 (22.64)	22 (25.00)	24 (34.29)^b^	11 (20.75)	13 (29.55)	20 (55.56)^c^
TIBC, µmol/L	43.33 ± 11.44	47.92 ± 9.40^c^	50.46 ± 9.63^c^	42.44 ± 12.03	42.18 ± 7.28^b^	45.25 ± 9.93^b^
Hemoglobin, g/L	87.84 ± 9.42	105.77 ± 13.13^c^	111.73 ± 17.87^c^	86.66 ± 10.72	99.99 ± 17.85^c^	106.39 ± 20.67^c^
LogEPO, mIU/mL^d^	0.77 ± 0.30	0.83 ± 0.42^b^	0.90 ± 0.42^c^	0.78 ± 0.31	1.52 ± 0.48^c^	1.56 ± 0.62^c^

Measurement data with a normal distribution are expressed as means ± standard deviation, and nonnormally distributed data are expressed as medians and interquartile range. SI denotes serum iron, sFt denotes serum ferritin, TSAT denotes transferrin saturation, TIBC denotes total iron-binding capacity

^a^Significant difference from baseline (*P* < 0.05)

^b^Significant difference between the groups (*P* < 0.05)

^c^Significant differences from baseline (*P* < 0.05) and between the groups (*P* < 0.05)

^d^The erythropoietin levels mentioned were measured before each administration

### Hemoglobin levels

**Fig. 4:**
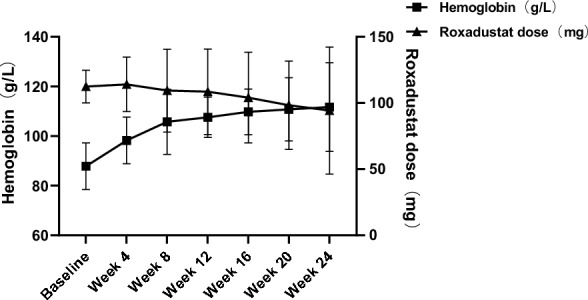
Hemoglobin level and roxadustat dose in roxadustat group

At baseline, the logEPO level was 0.77 ± 0.30 mIU/mL in the roxadustat group and 0.78 ± 0.31 mIU/mL in the ESA group. At week 24, the increase from baseline was 0.13 ± 0.42 mIU/mL and 0.78 ± 0.62 mIU/mL in the roxadustat and ESA groups. The difference between groups was -0.65 mIU/mL (95% CI, − 0.85 to − 0.45). Although the increase of logEPO levels in roxadustat group was much smaller than that in ESA group, no significant difference was observed in the improvement of anemia in PD patients between two groups. At week 24, hemoglobin levels increased 23.89 ± 17.87 g/L in roxadustat group and 19.73 ± 20.67 g/L in ESA group. The difference between groups was 4.16 g/L (95% CI, − 3.51 to 11.83). The percentage of patients with a hemoglobin level greater than or equal to 100 g/L but less than 120 g/L was 44.29% in the roxadustat group, and 47.22% in the ESA group in week 24 (Fig. 3B). The mean dose of roxadustat was 111.86 ± 12.77 mg at baseline, 106.71 ± 31.56 mg at week 8, and 94.29 ± 48.02 mg at week 24 (Fig. 4).

### Lipid metabolism levels

At week 24, the mean decreases in the lipid levels in the roxadustat group were as follows: 0.26 ± 0.98 mmol/L in the total cholesterol level (treatment difference, − 0.28 mmol/L; 95% CI, − 0.72 to 0.16), 0.39 ± 0.78 mmol/L in the low-density lipoprotein level (treatment difference, − 0.28 mmol/L; 95% CI, − 0.64 to 0.07), 0.10 ± 0.22 mmol/L in the high-density lipoprotein level (treatment difference, − 0.06 mmol/L; 95% CI, − 0.16 to 0.05), and 0.19 ± 0.55 mmol/L in the triglyceride level (treatment difference, − 0.59 mmol/L; 95% CI, − 1.07 to − 0.12).

### Adverse events and safety

The changes in the mean arterial pressure from baseline to week 24 were 1.67 ± 17.13 mmHg in the roxadustat group and 3.82 ± 11.68 mmHg in the ESA group (difference, − 2.14 mmHg; 95% CI, − 8.45 to 4.16). Although no difference was found in the proportion of patients who did not achieve the control goal (roxadustat, 64.29%; ESA, 69.44%), a higher proportion of patients in the ESA group increased or adjusted blood pressure medication (roxadustat, 25.47%; ESA, 47.17%).

Roxadustat did not increase the incidence of hyperkalemia (*P* = 0.651), and significantly reduced the incidence of metabolic acidosis (*P* < 0.001). The mean changes in the potassium levels were as follows: at week 8, a change of − 0.01 ± 0.62 mmol/L (95% CI, − 0.14 to 0.13) in the roxadustat group and − 0.25 ± 0.72 mmol/L (95% CI, − 0.47 to 0.03) in the ESA group; at week 24, a change of − 0.03 ± 0.59 mmol/L (95% CI, − 0.11 to 0.17) in the roxadustat group and − 0.14 ± 0.72 mmol/L (95% CI, − 0.38 to 0.11) in the ESA group. In the roxadustat group, 3.77% of patients had with hyperkalemia (> 5.50 mmol/L) at baseline, 2.27% at week 8, and 1.43% at week 24. The ESA group's percentages were 9.43% at baseline, 2.27% at week 8, and 8.33% at week 24. In the roxadustat group, the proportion of patients with bicarbonate levels less than 22 mmol was 48.11% at baseline, 26.41% at 8 weeks, and 18.57% at 24 weeks. The ESA group's percentage was 45.28% at baseline, 39.59% at week 8 and 30.56% at week 24.

High-sensitivity C-reactive protein levels in the roxadustat (*P* = 0.421) and ESA groups (*P* = 0.367) remained stable over the course of the study. 25 cases of peritonitis were identified during the 24-week study (roxadustat, 14; ESA, 11). The severity of peritonitis was mild/moderate and was resolved with antibiotic treatment, and no case resulted in the discontinuation of roxadustat and ESAs.

## Discussion

In this 24-week clinical study of PD patients, roxadustat significantly reduced the rise of sTFR and reduced the occurrence of functional iron deficiency by lowering hepcidin levels, although there was no significant difference in the incidence of absolute iron deficiency between the two groups. In addition, roxadustat improved anemia and lipid metabolism without increasing the incidence of hyperkalemia, metabolic acidosis, and peritonitis.

Since there were 22% of patients who had insufficient iron reserve state the number. The roxadustat group still had significantly improved iron metabolism levels in PD patients, with a smaller proportion of oral iron patients. We suggest that the decreased hepcidin levels and increased iron availability associated with roxadustat may have contributed to these findings [21]. Elevated hepcidin levels due to persistent inflammation [5] are often observed in patients with CKD [12, 22]. During the 24 weeks of the study, the hepcidin levels were significantly reduced in patients treated with roxadustat, resulting in reduced degradation of ferroportin (FPN) [23] and increased iron absorption and release to avoid the occurrence of functional iron deficiency [24]. The TIBC levels increased significantly in the roxadustat group but not in patients receiving ESAs, and this increase may be a direct result of stabilizing HIF levels with roxadustat. The gene-encoding transferrin is a HIF target with good properties because its enhancer region contains two HIF binding sites [25]. At week 8 of the study, the roxadustat group showed a more significant reduction in sFt than the ESA group, indicating increased iron utilization. In addition to its inhibitory effect on hepcidin, HIF also induces the expression of heme oxygenase-1, ceruloplasmin, FPN, and transferrin receptors [26], all of which are essential for iron cycling.

Clinically, TSAT (an index of iron utilization state) < 20% and sFt (an index of iron storage state) < 100 ng/mL is diagnosed as absolute iron deficiency in PD patients. Functional iron deficiency is characterized by TSAT < 20% and normal or elevated sFt levels [27]. Notably, the specificities of sFt and TSAT are very poor, and the results are affected by various factors, especially inflammation [7]. Therefore, to better analyze the iron metabolism level of patients, this parameter must be supplemented with high-sensitivity C-reactive protein and nutrition index tests. sTFR is mainly derived from the FPN on the juvenile erythroid membrane surface and mediates iron-containing transferrin into the cells [28]. Unlike other iron parameters, it is not affected by inflammation, trauma, stress or other factors [8, 9], and the sTFR concentration increases rapidly in the early stage of iron deficiency, leading to an early diagnosis of IDA [29]. The concentration of sTFR can reflect the demand of cells for iron and is a good indicator of functional iron deficiency [30, 31]. In recent years, many studies have shown that elevated sTFR levels were associated with the high prevalence of cardiovascular diseases [32, 33]. In the 24th week, although the proportion of patients with absolute iron deficiency did not increase significantly, the sTFR level increased significantly, suggesting that iron deficiency occurred in PD patients with the increase in iron utilization, and appropriate iron supplementation may be necessary.

Because roxadustat mediates the transition from aerobic to anaerobic metabolism, the immediate consequence of increased glycolysis is tissue acidification and lactic acid overproduction. The acidosis reaction leads to the release of potassium ions from cells, resulting in hyperkalemia [34]. In a phase III study conducted in China involving CKD patients on dialysis, patients treated with roxadustat were more likely to develop hyperkalemia compared with ESAs patients [35]. We found that the incidence of hyperkalemia and metabolic acidosis in this study was inconsistent with previous reports. We speculate that this may be related to the use of diuretics and bicarbonate supplements [36].

CKD patients often suffer from chronic inflammation due to excessive production and retention of urinary toxins, abnormal intestinal flora, and changes in the integrity of intestinal barrier. Inflammation blocks the output and absorption of iron that bacteria need to survive by stimulating the expression of hepcidin [37], which is thought to be a defense mechanism against infection [38]. In this study, roxadustat significantly reduced hepcidin levels without increasing high-sensitivity C-reactive protein level and the incidence of peritonitis.

It should be noted that this study has the following limitations: first of all, this trial is a single-center study, and these participants may not be generalizable to other populations. Secondly, we believe that the use of diuretics and bicarbonate supplements reduced the incidence of hyperkalemia and metabolic acidosis in roxadustat group, but we regret that the use of drug was not counted during the study. Finally, this study only studied the changes in patients’ condition after 24 weeks of treatment, which may not be long enough to observe the safety of roxadustat. The risks of angiogenesis and cancer associated with HIF have been demonstrated, and further studies are needed before firm conclusions can be drawn.

## Conclusion

In conclusion, this 24-week prospective cohort study comparing the efficacy of roxadustat and ESAs in PD patients showed the benefit of roxadustat in improving iron metabolism. In addition to its inhibitory effect on hepcidin, roxadustat also induces the expression of molecules required for iron circulation. Thus, the increased iron consumption observed in the roxadustat group may be attributable not only to enhanced hematopoietic production, but also to improved iron utilization efficiency. Information on the differences in the effects of roxadustat and ESAs on iron metabolism could help in selecting appropriate treatment options for PD patients. Roxadustat displayed a manageable safety with no increased risk of the incidence of hyperkalemia, metabolic acidosis and peritonitis.

## Data Availability

The data that support the findings of this study are available on request from the corresponding author.

## Abbreviations

CKD: Chronic kidney disease
ESAs: Erythropoiesis stimulating agents
EPO: Erythropoietin
FPN: Ferroportin
HIF-PHI: Hypoxia inducing factor-prolyl hydroxylase inhibitor
IDA: Iron deficiency anemia
PD: Peritoneal dialysis
SI: Serum iron
sFt: Serum ferritin
sTFR: Serum soluble transferrin receptor
TIBC: Total iron binding capacity
TSAT: Transferrin saturation
